# Association Between Egg Consumption and Dementia Risk in the EPIC-Spain Dementia Cohort

**DOI:** 10.3389/fnut.2022.827307

**Published:** 2022-02-23

**Authors:** Hernando J. Margara-Escudero, Raul Zamora-Ros, Izar de Villasante, Marta Crous-Bou, María-Dolores Chirlaque, Pilar Amiano, Javier Mar, Aurelio Barricarte, Eva Ardanaz, José María Huerta

**Affiliations:** ^1^Unit of Nutrition and Cancer, Cancer Epidemiology Research Programme, Catalan Institute of Oncology (ICO), Bellvitge Biomedical Research Institute (IDIBELL), Barcelona, Spain; ^2^Department of Epidemiology, Harvard T.H. Chan School of Public Health, Boston, MA, United States; ^3^Department of Epidemiology, Murcia Regional Health Council, Biomedical Research Institute of Murcia (IMIB-Arrixaca), Murcia, Spain; ^4^Centro de Investigación Biomédica en Red de Epidemiología y Salud Pública (CIBERESP), Madrid, Spain; ^5^Ministry of Health of the Basque Government, Sub-directorate for Public Health and Addictions of Gipuzkoa, San Sebastian, Spain; ^6^Public Health Division of Gipuzkoa, BioDonostia Research Institute, San Sebastian, Spain; ^7^Research Unit, Basque Health Service (Osakidetza), Debagoiena Integrated Healthcare Organisation, Arrasate-Mondragón, Spain; ^8^Group of Economic Evaluation of Chronic Diseases, Kronikgune Health Services Research Institute, Barakaldo, Spain; ^9^Group of Epidemiology of Cancer and Other Chronic Diseases, Navarra Public Health Institute, Pamplona, Spain; ^10^Group of Epidemiology of Cancer and Other Chronic Diseases, Navarra Institute for Health Research (IdiSNA), Pamplona, Spain

**Keywords:** dementia, egg, intake, Alzheimer, EPIC-Spain, cohort, Mediterranean diet

## Abstract

**Background:**

Current evidence suggests that egg composition might have potential neuroprotective effects. Our aim was to determine the association between egg consumption and the risk of dementia in a Mediterranean population.

**Methods:**

This study was carried out in 3 centers from the European Prospective Investigation into Cancer and Nutrition (EPIC)-Spain Dementia Cohort, i.e., 25,015 participants aged 30–70 years, recruited in 1992–1996, and followed up for a mean of 21.5 years.

**Results:**

A total of 774 incident dementia cases were diagnosed and validated, of which 518 were Alzheimer's disease (AD). Data on egg consumption were estimated using a validated dietary history questionnaire at recruitment. Cox proportional hazards models, adjusted for confounders, were used in the analyses. No association was observed between egg consumption and either total dementia [hazard ratio between extreme quartiles (HR_Q4vs.Q1_: 1.05; 95% CI 0.85–1.31; *p*-trend = 0.93)] or AD (HR_Q4vs.Q1_ 0.93; 95% CI 0.72–1.21; *p*-trend = 0.50) risks. After dividing the population by adherence to the relative Mediterranean diet (rMED) score, a borderline inverse association was found between egg intake and both total dementia (HR_Q4vs.Q1_: 0.52; 95% CI 0.30–0.90; *p*-trend = 0.10) and AD (HR_Q4vs.Q1_: 0.52; 95% CI 0.27–1.01; *p*-trend = 0.13) risks within participants with low adherence to rMED score. However, no association was observed in participants with medium and high adherence to rMED score.

**Conclusion:**

This prospective study suggests that egg consumption is associated with a reduced risk of dementia, and specifically of AD, in the adult population with low adherence to rMED score; whereas it has no impact in subjects with moderate and high MD adherence.

## Introduction

Dementia is commonly defined as a progressive cognitive decline that affects multiple cognitive domains causing a decline in independence and daily functions (1). Alzheimer's disease (AD) is the most common cause of dementia, which accounts for 60–70% of cases (2). The prevalence of dementia varies across geographical regions, although overall, it increases consistently due to population aging (2, 3). Currently, it impacts ~50 million people's lives worldwide and this number is expected to rise up to 152 million by 2050 (3, 4). There is still no effective treatment for dementia; therefore, primary prevention is the most efficient strategy to reduce the incidence rates/burden of the disease. Modifying 12 known modifiable risk factors, such as education, smoking, obesity, alcohol, physical activity, and diet, might prevent or delay up to 40% of dementias (4).

On the one hand, eggs are of particular nutritional interest, as they are rich in essential amino acids, unsaturated fatty acids, all B vitamins, folate, fat-soluble vitamins, and minerals, such as phosphorus, selenium, iron, iodine, and zinc (5, 6). Furthermore, eggs are a widely available source of bioactive compounds, such as choline, lutein, zeaxanthin, and other essential nutrients, that might have been reported to have a positive impact on health, particularly, on cognition (6). On the other hand, egg yolk is a major source of dietary cholesterol and hypercholesterolemia, which has been reported as a risk factor for neurodegenerative diseases (7). However, egg's cholesterol is not well absorbed and does not mainly affect blood levels (8).

After considering a large number of existing prospective studies, a recent umbrella meta-analysis concluded that there was mainly no association between egg consumption and several health outcomes, such as cancer, cardiovascular and metabolic diseases (9). However, the current epidemiological evidence on neurodegenerative diseases is still scarce and inconclusive. Our previous study in a Mediterranean prospective cohort (European Prospective Investigation into Cancer [EPIC]-Spain) showed that moderate egg consumption was associated with a 10% risk reduction of mortality from neurodegenerative diseases (10). Moreover, a prospective cohort of community old people living in China observed an inverse relation between egg consumption and cognitive decline (11). However, in a prospective Finish cohort, no association between egg consumption and risk of incident dementia was found (12). Hence, we aimed to assess the relationships between egg consumption and dementia risk, particularly, AD, in the EPIC-Spain Dementia Cohort, a Mediterranean study with a wide variability in egg consumption (10).

## Materials and Methods

### Subjects and Study Design

European Prospective Investigation into Cancer is a multi-national prospective cohort that includes 23 collaborating centers in ten European countries (13, 14). The EPIC study was approved by the local ethics committees in the participating centers and the Medical Ethical Committee of the Bellvitge University. All participants signed a written informed consent at recruitment. The EPIC-Spain cohort enrolled 41,437 adult volunteers from 5 Spanish regions: Asturias, Granada, Gipuzkoa, Murcia, and Navarra (15).

In the present study, we used data from the EPIC-Spain Dementia Cohort, which includes three Spanish centers with available data on incident dementia: Gipuzkoa, Navarra, and Murcia. The study sample consists of 25,015 participants aged 30–70 years at recruitment between 1992 and 1996 (15). At baseline, all participants were apparently healthy, mostly blood donors (60%).

### Dietary Assessment and Lifestyle Data

Data on habitual food intake during the previous 12 months were collected at enrollment using a dietary history questionnaire that had been previously validated in Spain (16). The questionnaire was open, but was structured by meals and included a list of 662 common foods and recipes from each region. Recipes were broken down into ingredients. All foods consumed at least twice a month were registered. The questionnaire includes data on food preparation, frequency of consumption, and portion size of each food item consumed (g/day). The portion size of each food item was determined by several methods: a set of 35 photographs, natural units, and household measurements (15). The egg consumption variable represents total egg consumption (g/day), considering 50 g as a standard egg. In the descriptive analysis, egg consumption was considered in g/day. To facilitate the interpretation of the results, in the survival models, the egg consumption was considered as eggs per week (equivalent to 50 g/week or 7.1 g/day). The EPIC Nutrient DataBase was used to determine daily total energy (kcal/day) and nutrient intake (17).

Additional questionnaires were used to collect information on sociodemographic and lifestyle factors (15), such as educational level, tobacco consumption, physical activity (18), reproductive, and medical history. Anthropometric measures, such as weight and height, were measured by trained nurses at the recruitment. Body mass index (BMI) was calculated as weight (kg) divided by squared height (m^2^) (15).

### Mediterranean Diet Score

The relative Mediterranean diet (rMED) score (0–18 points) was used to measure the level of adherence to the Mediterranean dietary pattern (19). Briefly, the rMED score is based on 9 components: 6 considered as positive (fruit, vegetables, olive oil, legumes, fish, and cereals), and 2 presumed not to reflect the Mediterranean diet (meat and dairy products). Each rMED component (apart from alcohol) was measured as g per 2,000 kcal/day (to express intake as energy density) and was divided into tertiles of dietary intake. Finally, alcohol was scored dichotomously, assigning 2 points for moderate consumers (5–25 g/d for women and 10–50 g/d for men) and 0 for subjects outside (above or below) the sex-specific range. The rMED score was evaluated as a continuous variable and as a categorical variable (low adherence, 0–6 units; medium adherence, 7–10 units; and high adherence, 11–18 units).

### Ascertainment of Dementia Cases

Case ascertainment was organized in two phases and was previously described (20). In phase I, potential cases were identified by linkage of the EPIC-Spain database and all available health databases that could contain dementia-related clinical information. During phase II, validation of incident cases of dementia was assessed by a group of neurologists after a careful evaluation. The subtype of dementia, such as AD, was determined when competently detailed clinical information about subtypes was available in medical reports. Even though participants in the EPIC study did not undergo baseline cognitive assessment, subjects were demanded to be mentally and physically able to realize extensive questionnaires, which are required to guarantee a normal cognition (20). The last complete vital status check was in December 2017 for Gipuzkoa, December 2015 for Navarra, and November 2016 for Murcia through record linkage with the Spanish National Death Index.

### Statistical Analyses

Hazard ratio (HR), and the corresponding 95% CI, for the associations between egg consumption and dementia risk was estimated using a multivariable Cox proportional hazards models. The proportional hazard's assumption was analyzed using Schoenfeld residuals. The primary time variable was defined as age at recruitment, exit time was the age at diagnosis for dementia cases, and age at death or censoring for non-cases. For all analyses, we used four models with increasing degrees of adjustment to account for potential confounding factors. The first model was stratified by age at recruitment (<40, 40–44, 45–49, 50–54, 55–59, and ≥60), sex, and center. The second model was additionally adjusted for socio-demographic and lifestyle covariables: educational level (none, primary, technical school, secondary, university, or higher), smoking status (never, former, and current), physical activity (inactive, moderately inactive, moderately active, and active), BMI (kg/m^2^), and alcohol consumption (g/day). The third model was additionally adjusted for prevalent self-reported chronic diseases: diabetes, hypertension, stroke, ischemic heart disease, and cancer. The fourth model was further adjusted for dietary variables: total energy intake (kcal/day) and rMED score (continuous) variables. Egg consumption was analyzed as quartiles, sex-specific quartiles, and as a continuous variable (per 1 egg/week). Tests for trend were calculated using quartile-based scores 1–4 used as continuous variables. Separate models were defined to assess the risk of dementia by subtypes (AD vs. non-AD). However, we did not present results on non-AD cases due to the low number of cases and the heterogeneity between the different pathologies included in this subtype.

Effect modification by sex, center, age at recruitment (<45 years, 45–54 years, and ≥55 years), BMI (<25, 25–29.9, ≥30 kg/m^2^), smoking status (never, former, and current), and adherence to rMED score (low, medium, and high) were investigated by modeling the multiplicative interaction term between each of these separate variables and egg consumption and tested using the log-likelihood ratio test. Separate models by rMED adherence score were fitted because a significant interaction between adherence to rMED categories and egg intake was detected.

Sensitivity analyses were conducted, excluding (1) dementia cases diagnosed in the first 5 years of follow-up to control for the possibility of reverse causality (*n* = 11), (2) subjects with at least one major chronic prevalent pathology at recruitment: cancer, diabetes, ischemic heart disease, and stroke (*n* = 1,650), (3) energy intake miss-reporters; which are those with a reported energy intake beyond 30% of the estimated energy requirement (corresponding to a cutoff of 2 SD of the ratio of estimated energy intake to predicted total energy expenditure) (*n* = 7,678), and (4) participants with prevalent self-reported chronic diseases and/or energy intake miss-reporters (*n* = 8,855). The software program used to analyze the data was R 3.2.1 software (R Foundation for Statistical Computing, Vienna, Austria).

## Results

A total of 25,015 participants, (43%) men and (57%) women, were followed up for a mean of 21.5 years. Within this period, 774 dementia cases were identified, 518 of which were recorded as AD, and 256 as non-AD. Participants in the highest quartile of egg consumption were more likely to be from northern Spanish centers (Navarra and Gipuzkoa), men, younger, slightly more overweight, physically active, current smokers, consume more total energy and alcohol have slightly less adherence to an rMED score and less prevalent chronic diseases compared to those in the lowest quartile (Table 1).

**Table 1:** Baseline characteristics of the studied population by quartiles of egg consumption (g/day) in the EPIC-Spain Dementia Cohort.

**Baseline characteristics**	**Q1**	**Q2**	**Q3**	**Q4**
Cuts-off of egg consumption, g/day	0, 13.2	13.3, 24.1	24.2, 37.8	37.9, 248
*N*	6,255	6,253	6,253	6,254
**Center**, ***N*** **(%)**				
Murcia	3,168 (50.6)	2,579 (41.2)	1,814 (29.0)	954 (15.3)
Navarra	1,504 (24.0)	1,774 (28.4)	2,109 (33.7)	2,697 (43.1)
Gipuzkoa	1,583 (25.3)	1,900 (30.4)	2,330 (37.3)	2,603 (41.6)
Sex, female, *N* (%)	4,074 (65.1)	4,088 (65.4)	3,718 (59.5)	2,386 (38.2)
Age at recruitment years [median (IQR)]	50.7 (44.2, 57.6)	48.4 (42.3, 55.2)	48.1 (42.5, 54.4)	48.3 (43.2, 54.5)
BMI, kg/m^2^, median (IQR)	27.6 (25.1, 30.5)	27.4 (24.9, 30.3)	27.4 (25, 30.2)	28.0 (25.6, 30.6)
Alcohol, g/d, median (IQR)	2.7 (0, 15.3)	3.5 (0, 17.9)	5.1 (0, 22.1)	13.4 (0.64, 37.2)
Total energy, kcal/d, median (IQR)	1,934 (1,563, 2,387)	2,104 (1,715, 2,543)	2,263 (1,846, 2,724)	2,552 (2,065, 3,090)
**Educational level**, ***N*** **(%)**				
None	2,464 (39.4)	2,021 (32.3)	1,891 (30.2)	1,718 (27.5)
Primary school	2,153 (34.4)	2,499 (40.0)	2,672 (42.7)	2,860 (45.7)
Technical training	469 (7.5)	551 (8.8)	599 (9.6)	691 (11.0)
Secondary school	345 (5.5)	378 (6.0)	381 (6.1)	399 (6.4)
University degree	795 (12.7)	775 (12.4)	683 (10.9)	552 (8.8)
**Physical activity**, ***N*** **(%)**				
Inactive	2,396 (38.3)	2,255 (36.1)	2,103 (33.6)	1,695 (27.1)
Moderately inactive	2,123 (33.9)	2,147 (34.3)	2,183 (34.9)	1,969 (31.5)
Moderately active	1,083 (17.3)	1,172 (18.7)	1,177 (18.8)	1,640 (26.2)
Active	653 (10.4)	679 (10.9)	790 (12.6)	950 (15.2)
**Smoking status**, ***N*** **(%)**				
Never	3,519 (56.3)	3,544 (56.7)	3,329 (53.2)	2,797 (44.7)
Former	1.154 (18.4)	1,053 (16.8)	1,094 (17.5)	1,231 (19.7)
Current	1,579 (25.2)	1,651 (26.4)	1,827 (29.2)	2,222 (35.5)
Diabetes, case, *N* (%)	407 (6.5)	314 (5.0)	261 (4.2)	264 (4.2)
Hypertension, case, *N* (%)	1,454 (23.2)	1,230 (19.7)	1,183 (18.9)	1,159 (18.5)
Stroke, case, *N* (%)	18 (0.3)	15 (0.2)	14 (0.2)	17 (0.3)
Ischemic heart disease, case, *N* (%)^*^	70 (1.1)	50 (0.8)	26 (0.4)	34 (0.5)
Cancer, case, *N* (%)	64 (1.0)	54 (0.9)	59 (0.9)	44 (0.7)
**Adherence to relative Mediterranean diet**, ***N*** **(%)**				
Low	1,269 (20.3)	1,255 (20.1)	1,413 (22.6)	1,508 (24.1)
Medium	3,111 (49.7)	3,304 (52.8)	3,283 (52.5)	3,364 (53.8)
High	1,875 (30.0)	1,694 (27.1)	1,557 (24.9)	1,382 (22.1)

*IQR, interquartile range. Unknown's values in Q1: education level (0.5%), smoking status (0.0%), hypertension (0.2%), diabetes (0.1%), stroke (0.9%), and cancer (0.8%). Unknown's values in Q2: education level (0.5%), smoking status (0.1%), hypertension (0.2%), diabetes (0.1%), stroke (1.0%), and cancer (0.6%). Unknown's values in Q3: education level (0.4%), smoking status (0.0%), hypertension (0.1%), diabetes (0.1%), stroke (1.0%), and cancer (0.6%). Unknown's values in Q4: education level (0.5%), smoking status (0.1%), hypertension (0.3%), diabetes (0.12%), stroke (1.6%), and cancer (0.5%)*.

* *In ischemic heart disease, the unknown values were included as non-cases due to the low number of cases (N = 2)*.

In the fully adjusted Cox models, no association between egg consumption and dementia risk was detected analyzing either extreme quartiles (HR_Q4vs.Q1_: 1.05; 95% CI 0.85–1.31; *p*-trend = 0.93) or the continuous variable (HR_egg/week_: 1.06; 95% CI 0.96–1.16; Table 2). Similar results were observed for sex-specific quartiles with dementia (HR_Q4vs.Q1_: 1.06; 95% CI 0.92–1.22; *p*-trend = 0.36). Non-statistically significant results were observed investigating associations between egg consumption and AD risk (HR_Q4vs.Q1_: 0.93; 95% CI 0.72–1.21; *p*-trend = 0.50). The results from the sensitivity analyses, excluding dementia cases in the first 5 years, participants with at least one major chronic prevalent pathology at recruitment, and energy miss-reporters, were almost identical to those within the entire cohort (Supplementary Table 1).

**Table 2:** Adjusted hazard ratios (HRs) and 95% CI for dementia according to egg consumption in the EPIC-Spain Dementia Cohort.

	**Q1** ** HR (95% CI)**	**Q2** ** HR (95% CI)**	**Q3** ** HR (95% CI)**	**Q4** ** HR (95% CI)**	***P*-trend**	**Continuous (eggs/week)**
Dementia (cases)	254	191	155	174	774	774
Model 1	1 (ref)	0.96 (0.80, 1.17)	0.87 (0.71, 1.07)	1.05 (0.85, 1.29)	0.99	1.05 (0.96, 1.15)
Model 2	1 (ref)	0.96 (0.79, 1.16)	0.85 (0.69, 1.05)	1.01 (0.82, 1.25)	0.75	1.04 (0.95, 1.14)
Model 3	1 (ref)	0.96 (0.79, 1.16)	0.87 (0.70, 1.06)	1.04 (0.84, 1.28)	0.90	1.05 (0.95, 1.15)
Model 4	1 (ref)	0.97 (0.80, 1.17)	0.88 (0.71, 1.08)	1.05 (0.85, 1.31)	0.93	1.06 (0.96, 1.16)
Alzheimer (cases)	178	118	109	113	518	518
Model 1	1 (ref)	0.83 (0.66, 1.05)	0.84 (0.66, 1.08)	0.93 (0.72, 1.20)	0.50	1.00 (0.89, 1.11)
Model 2	1 (ref)	0.83 (0.65, 1.05)	0.83 (0.65, 1.06)	0.91 (0.71, 1.18)	0.48	0.99 (0.88, 1.11)
Model 3	1 (ref)	0.83 (0.66, 1.06)	0.84 (0.66, 1.07)	0.94 (0.73, 1.21)	0.38	1.00 (0.89, 1.12)
Model 4	1 (ref)	0.83 (0.66, 1.06)	0.84 (0.65, 1.07)	0.93 (0.72, 1.21)	0.50	1.00 (0.89, 1.12)

*Model 1 stratified by center, age at recruitment in 5 years categories, and sex. Model 2 additionally adjusted for educational level, physical activity, smoke status, BMI (kg/m^2^), and alcohol intake (g/day). Model 3 additionally adjusted for diabetes, hypertension, stroke, ischemic heart disease, and cancer. Model 4 additionally adjusted for energy intake (kcal/day) and adherence to relative Mediterranean diet score*.

No significant effect modification on the relationship between egg consumption and dementia risk was detected across categories of the investigated variables, except for the rMED score. A borderline inverse association between egg intake and dementia risk in the low rMED adherence category (HR_Q4vs.Q1_: 0.52; 95% CI 0.30–0.90; *p*-trend = 0.10) was observed, but not in the rest of the rMED score categories (medium and high rMED adherence score; Table 3). Similarly, a borderline inverse association was only detected with AD risk (HR_Q4vs.Q1_: 0.52; 95% CI 0.27–1.01; *p*-trend = 0.13) in the low rMED adherence category.

**Table 3:** Adjusted hazard ratios (HRs) and 95% CI for the interaction between relative Mediterranean diet (rMED) score and egg consumption in the EPIC-Spain Dementia Cohort.

rMED score/Categories^*^ (Dementia)	**Q1** ** HR (95% CI)**	**Q2** ** HR (95% CI)**	**Q3** ** HR (95% CI)**	**Q4** ** HR (95% CI)**	***P*-trend**	**Continuous (eggs/week)**
Low Adherence (Cases)	57	30	30	23	140	140
Cuts-off egg consumption (g/day)	0–14.1	14.2–25.7	25.8–39.7	39.8–248		
Model 1	1 (ref)	0.70 (0.44, 1.10)	0.73 (0.46, 1.15)	0.56 (0.33, 0.96)	0.18	0.85 (0.67, 1.07)
Model 4	1 (ref)	0.69 (0.44, 1.10)	0.69 (0.43, 1.11)	0.52 (0.30, 0.90)	0.10	0.81(0.64, 1.04)
Medium Adherence (cases)	129	116	79	95	419	419
Cuts-off egg consumption (g/day)	0–13.8	13.9–24.6	24.7–38.3	38.4–200		
Model 1	1 (ref)	1.13 (0.88, 1.46)	0.84 (0.63, 1.13)	1.11 (0.83, 1.48)	0.23	1.07 (0.95, 1.21)
Model 4	1 (ref)	1.16 (0.90, 1.51)	0.89 (0.66, 1.19)	1.20 (0.89, 1.62)	0.08	1.11 (0.98, 1.26)
High adherence (Cases)	64	51	48	52	168	168
Cuts-off egg consumption (g/day)	0–11.7	11.8–21.6	21.7–35.1	35.2–190		
Model 1	1 (ref)	0.79 (0.54, 1.14)	0.74 (0.50, 1.08)	0.83 (0.56, 1.21)	0.93	0.99 (0.83, 1.17)
Model 4	1 (ref)	0.87 (0.59, 1.26)	0.83 (0.56, 1.23)	0.93 (0.61, 1.39)	0.79	1.02 (0.85, 1.21)
rMED score/Categories^*^ (Alzheimer)	**Q1** **HR (95% CI)**	**Q2** **HR (95% CI)**	**Q3** **HR (95% CI)**	**Q4** **HR (95% CI)**	***P*** **-trend**	**Continuous (eggs/week)**
Low Adherence (cases)	38	20	17	15	90	90
Cuts-off egg consumption (g/day)	0–14.1	14.2–25.7	25.8–39.7	39.8–248		
Model 1	1 (ref)	0.68 (0.39, 1.19)	0.58 (0.32, 1.05)	0.53 (0.28, 1.01)	0.14	0.79 (0.59, 1.07)
Model 4	1 (ref)	0.70 (0.40, 1.22)	0.56 (0.30, 1.02)	0.52 (0.27, 1.01)	0.13	0.78 (0.57, 1.06)
Medium Adherence (cases)	94	72	58	61	285	285
Cuts-off egg consumption (g/day)	0–13.8	13.9–24.6	24.7–38.3	38.4–200		
Model 1	1 (ref)	0.96 (0.70, 1.31)	0.85 (0.61, 1.19)	0.99 (0.70, 1.40)	0.66	1.03 (0.88, 1.19)
Model 4	1 (ref)	0.99 (0.72, 1.35)	0.87 (0.62, 1.23)	1.04 (0.72, 1.49)	0.47	1.05 (0.90, 1.23)
High adherence (cases)	45	30	31	37	102	102
Cuts-off egg consumption (g/day)	0–11.7	11.8–21.6	21.7–35.1	35.2–190		
Model 1	1 (ref)	0.64 (0.40, 1.02)	0.63 (0.40, 1.01)	0.76 (0.48, 1.20)	0.43	0.91 (0.73, 1.13)
Model 4	1 (ref)	0.71 (0.44, 1.14)	0.70 (0. 43, 1.13)	0.83 (0.51, 1.36)	0.54	0.93 (0.74, 1.16)

* *Low adherence, 0–6; medium adherence, 7–10; and high adherence, 11–18. Model 1 stratified by center, age at recruitment in 5 years categories, and sex. Model 4 additionally adjusted for educational level, physical activity, smoke status, BMI (kg/m^2^), alcohol intake (g/day), diabetes, hypertension, stroke, ischemic heart disease, cancer, and energy intake (kcal/day)*.

## Discussion

In this prospective Mediterranean cohort of 25,015 adult participants, we found that egg consumption was not associated with risk of dementia in general or specifically with AD risk. However, in participants with low adherence to rMED score, borderline inverse associations between egg intake and risk of both dementia and AD were observed, but not within those with medium or high adherence to rMED score.

To our knowledge, this is the first study that analyzes the association between egg consumption and the incidence of dementia in a Mediterranean cohort. In the present study, we did not observe a protective relation against dementia risk in all subjects. However, in a previous study, in the EPIC-Spain cohort, we found that moderate egg consumption was associated with a 10% lower mortality risk from neurodegenerative diseases that include mostly mortality cases from dementia but also from Parkinson's disease (10). Similarly, egg intake was associated with lower mortality from AD in a US large prospective cohort (21). In addition, the Chinese Longitudinal Health Longevity Study showed an inverse association with cognitive decline (11).

Eggs provide bioactive compounds, such as lutein, choline, zeaxanthin, and high-value proteins, that may have a protective role against dementia due to their beneficial effects on inflammation (22, 23). It has been suggested that lutein may help to protect brain tissues from the accumulated effects of oxidative and inflammatory stress (24). Dietary choline intake in adults may also influence cognitive function via an effect on phosphatidylcholine; polyunsaturated species of phosphatidylcholine the levels of which are reduced in brains from patients with AD and is associated with higher memory performance and resistance to cognitive decline (25). Furthermore, choline via its metabolites participates in pathways that regulate methylation of genes related to memory and cognitive functions at different stages of development (26). Thus, a cohort in Finland observed an inverse association between dietary phosphatidylcholine intake and dementia risk (27). Eggs are the main food source of phosphatidylcholine in Western diets (27).

In our study, the protective association was only observed in participants with low adherence to the rMED score. A potential explanation is that in subjects with lower rMED adherence score, eggs might be the major food source of some bioactive compounds (such as lutein, choline, zeaxanthin, and proteins) with neuroprotective effects. However, in participants with medium and high adherence to rMED score, these protective compounds, and others (such as folate, vitamin E, vitamin C, ß-carotene, and polyphenols) (28, 29), could be provided by other food sources, such as fruits and vegetables, especially cruciferous vegetables, dark and green leafy vegetables, root vegetables, cabbage, and tomatoes (30–32). Nuts also seem to have a protective impact on individuals with a higher risk of cognitive impairment (33). Moreover, legumes and vegetal oils could improve insulin sensitivity, which could, in turn, influence cognitive function (34). Indeed, in the EPIC-Spain Dementia Cohort, a recent investigation observed that a high rMED adherence score was associated with a 20% lower risk of dementia (35).

The finding that some specific contents of the diet are associated with a reduced dementia risk is key in boosting the primary prevention, which aims to delay the pathological changes occurring in the brain that determines the development of dementia. As dementia management has evolved to focus on various primary prevention strategies in healthy individuals (36), the better the contents of these strategies are identified, the greater their effectiveness. Predictive models use midlife risk factors to measure their association with dementia risk in older adults (37, 38). The added value of our work relies on the assessment of a new potential protective factor, egg intake that may contribute to improve these prediction models and dietary prevention strategies.

The current study has some limitations. Our results may be influenced by measurement errors that may have attenuated our findings, although a validated dietary history questionnaire was used (17). Dietary exposures and lifestyle variables were measured only at baseline, and therefore, we cannot evaluate changes during the follow-up. Dietary modifications in the years before dementia or after chronic disease ascertainment are probable, but the exclusions of either dementia cases that occurred in the first 5 years of the follow-up or prevalent cases of chronic diseases at recruitment did not modify our findings. In addition, there is the possibility of a sub-registration of dementia cases that may have not been considered in the public health system due to lack of consultation in public centers, emigration, or premature death for other reasons, although we estimate this probability as low (39). Finally, a misclassification of the cases might be possible; however, all incident dementia cases were validated by neurologists. The strengths of this study are its prospective design, long follow-up, relatively large number of dementia cases, and the wide range of egg intake across participants from three Spanish regions with standardized information on diet and lifestyle exposures.

## Conclusions

In conclusion, this prospective study suggests that a higher intake of whole eggs was associated with a lower risk of dementia in adult-population that had a lower adherence to rMED score; whereas it has no impact in subjects with moderate and high rMED adherence scores. Further studies are required to evaluate this association in other populations with different dietary patterns.

## Data Availability Statement

The data analyzed in this study is subject to the following licenses/restrictions: The data will be made available from the authors upon reasonable request to EPIC-Spain Dementia Cohort. Requests to access these datasets should be directed to Jose María Huerta, jmhuerta.carm@gmail.com.

## Ethics Statement

The studies involving human participants were reviewed and approved by Medical Ethical Committee of the Bellvitge University. The patients/participants provided their written informed consent to participate in this study.

## Author Contributions

RZ-R and JH contributed to the conceptualization. MC-B, M-DC, PA, JM, AB, EA, and JH contributed to data resources. HM-E and IV participated in statistical analysis. HM-E contributed to writing—original draft preparation. RZ-R, MC-B, and JH contributed to writing—review and editing. All authors have read and agreed to the final version of the manuscript.

## Funding

The EPIC-Spain is financially supported by the Health Research Fund (FIS)-Instituto de Salud Carlos III (ISCIII), Regional Governments of Andalucía, Asturias, Basque Country, Murcia and Navarra, and the Catalan Institute of Oncology (ICO). We also thank the CERCA Program / Generalitat de Catalunya for the institutional support to IDIBELL. RZ-R would like to thank the Miguel Servet program (CPII20/00009) from the Institute of Health Carlos III (Spain) and the European Social Fund (ESF).

## Conflict of Interest

The authors declare that the research was conducted in the absence of any commercial or financial relationships that could be construed as a potential conflict of interest.

## Publisher's Note

All claims expressed in this article are solely those of the authors and do not necessarily represent those of their affiliated organizations, or those of the publisher, the editors and the reviewers. Any product that may be evaluated in this article, or claim that may be made by its manufacturer, is not guaranteed or endorsed by the publisher.

## Abbreviations

AD: Alzheimer's disease
BMI: body mass index
CI: confidence interval
EPIC: European Prospective Investigation into Cancer
HR: hazard ratio
rMED: relative Mediterranean diet.
