# Supplementary material for: Association Between Egg Consumption and Dementia Risk in the EPIC-Spain Dementia Cohort
Source: Front Nutr. 2022 Feb 23;9:827307. doi: 10.3389/fnut.2022.827307 (PMC8906465; doi:10.3389/fnut.2022.827307)
Supplement: Supplementary file 1 [file Table_1.DOCX]

Supplementary Material

# Supplementary Table 1. Sensitivity analyses for the association between egg consumption and dementia risk in EPIC-Spain Dementia Cohort

|  | **Q1**  **HR (95%CI)** | **Q2**  **HR (95% CI)** | **Q3**  **HR (95% CI)** | **Q4**  **HR (95% CI)** | **P-trend** | **Continuous**  **(eggs/week)** |
| --- | --- | --- | --- | --- | --- | --- |
| **Excluding cases with ≤5 years of follow-up (cases)** | **247** | **189** | **155** | **172** | **763** | **763** |
| Model 1 | 1 (ref) | 0.93  (0.76, 1.15) | 0.92  (0.74, 1.14) | 0.84  0.67, 1.04) | 0.90 | 1.06  (0.97, 1.16) |
| Model 4 | 1 (ref) | 1.10  (0.89, 1.36) | 1.09  (0.88 1.35) | 1.18  (0.94, 1.47) | 0.86 | 1.07  (0.97, 1.17) |
| **Excluding chronic disease (cases)** | **203** | **172** | **142** | **148** | **665** | **665** |
| Cutoff egg consumption | 0-13.5 | 13.6-24.4 | 24.5-38.1 | 38.2-248 |  |  |
| Model 1 | 1 (ref) | 1.08  (0.88, 1.33) | 0.97  (0.78, 1.21) | 1.07  (0.85, 1.34) | 0.75 | 1.01  (0.87, 1.17) |
| Model 4 | 1 (ref) | 1.09  (0.88, 1.34) | 0.96  (0.77, 1.21) | 1.05  (0.83, 1.33) | 0.89 | 1.06  (0.96, 1.17) |
| **Excluding energy miss-reporters (cases)** | **173** | 132 | 99 | 127 | 531 | 531 |
| Cutoff egg consumption | 0-12.7 | 12.8-23.1 | 23.2-36.2 | 36.3-200 |  |  |
| Model 1 | 1 (ref) | 1.00  (0.80, 1.26) | 0.80  (0.62, 1.03) | 1.14  (0.89, 1.45) | 0.76 | 1.08  (0.97, 1.20) |
| Model 4 | 1 (ref) | 1.00  (0.79, 1.25) | 0.79  (0.61, 1.02) | 1.10  (0.86, 1.41) | 0.86 | 1.06  (0.95, 1.19) |
| **Excluding energy miss-reporters + Chronic disease (cases)** | **145** | **115** | **94** | **110** | **464** | **464** |
| Cutoff egg consumption | 0-12.9 | 13-23.2 | 23.3-36.4 | 36.5-200 |  |  |
| Model 1 | 1 (ref) | 1.12  (0.87, 1.44) | 0.91  (0.70, 1.20) | 1.16  (0.89, 1.52) | 0.54 | 1.07  (0.95, 1.20) |
| Model 4 | 1 (ref) | 1.12  (0.87, 1.43) | 0.90  (0.69, 1.18) | 1.11  (0.85, 1.46) | 0.76 | 1.05  (0.93, 1.18) |
| **Excluding energy miss-reporters + chronic diseases + AD (cases)** | **92** | **80** | **64** | **72** | **308** | **308** |
| Cutoff egg consumption | 0-12-9 | 13-23.2 | 23.3-36.4 | 36.5-200 |  |  |
| Model 1 | 1 (ref) | 1.14  (0.84, 1.55) | 0.92  (0.66, 1.27) | 1.10  (0.79, 1.53) | 0. | 1.03  (0.89, 1.19) |
| Model 4 | 1 (ref) | 1.13  (0.83, 1.54) | 0.89  (0.64, 1.24) | 1.05  (0.75, 1.46) | 0.91 | 1.00  (0.87, 1.16) |

AD Alzheimer’s disease
